# Effect of tumor necrosis factor-α induced protein 8 like-2 on immune function of dendritic cells in mice following acute insults

**DOI:** 10.18632/oncotarget.8398

**Published:** 2016-03-28

**Authors:** Ying-yi Luan, Ren-qi Yao, Sen Tong, Ning Dong, Zhi-yong Sheng, Yong-ming Yao

**Affiliations:** ^1^ Medical School of Chinese PLA, The Chinese PLA General Hospital, Beijing, People's Republic of China; ^2^ Trauma Research Center, First Hospital Affiliated to The Chinese PLA General Hospital, Beijing, People's Republic of China; ^3^ 10th Student Team, Undergraduate Medical School, Second Military Medical University, Shanghai, People's Republic of China; ^4^ State Key Laboratory of Kidney Disease, The Chinese PLA General Hospital, Beijing, People's Republic of China

**Keywords:** dendritic cells, tumor necrosis factor-α induced protein 8 like-2, maturation, differentiation, Immunology and Microbiology Section, Immune response, Immunity

## Abstract

Tumor necrosis factor-α induced protein 8 like-2 (TNFAIP8L2, TIPE2) is a lately discovered negative regulator of innate immunity and cellular immunity. The present study was designed to investigate whether naturally occurring dendritic cells (DCs) could express TIPE2 mRNA/protein and its potential significance. Expressions of co-stimulatory molecules on DC surface and cytokines were analyzed to assess the functional role of TIPE2 in controlling DC maturation as well as activation. The activated DCs were assessed for their capacity to stimulate the proliferation and differentiation of T cells. It was found that TIPE2 was a cytoplasmic protein expressed in DCs, and the percentage of DCs which expressed co-stimulatory molecules and cytokines were obviously up-regulated when TIPE2 gene silenced by siRNA *in vitro* and *in vivo*. DCs undergone TIPE2 knockdown were found to promote the maturation of DCs, T-cell proliferation as well as differentiation, and they were significantly elevated IL-2 level and intranuclear NF-AT activation. Conversely, in over-expressing TIPE2 DC cells, it could inhibit T-cell proliferation and differentiation, and markedly down-regulate IL-2 expression and intranuclear NF-AT activation after scald injury. The results suggested that TIPE2 appeared to be a critical immunoregulatory molecule which affected DC maturation and subsequent T-cell mediated immunity.

## INTRODUCTION

Severe burn injury induces a temporal shift in immune reactivity that may induce septic syndrome or even death. The immune system responds to injury by means of rapidly producing early and late inflammatory cytokines, and also affecting maturation and differentiation of dendritic cells (DCs), as well as suppression of T cell function [1–3]. Despite the evolution of therapeutic strategies such as aggressive surgical techniques, extensive methods of supportive care, unfortunately, they have failed to reduce mortality significantly in severely septic patients after burns in many countries [4–6]. Therefore, it is of great significance to further elucidate the pathophysiological mechanisms, and to seek scientific interventional approaches for prevention and rational treatment of severe sepsis secondary to severe trauma or burns.

DCs are crucial in pathogen recognition and induction of specific immune responses to eliminate pathogens from the infected host, therefore they are professional antigen presenting cells (APCs) specialized in the capturing, processing, and transporting the antigen to lymphoid organs from the secondary lymphoid tissue [7–9]. The maturation of the DC has been implicated as the bridge plank between the innate and adaptive immune systems. During severe trauma, burns, and sepsis, DCs are stimulated to mature and present processed antigens *via* major co-stimulatory molecules (CD80 and CD86) and major histocompatibility complex (MHC) class II, and they engender the differentiation of different clones of T-helper (Th) cells [10, 11]. Furthermore, high mobility group box-1 protein (HMGB1), described as a late inflammatory cytokine, was recently proved to be potent stimulus for activation of DCs. Therefore, in the vitro study, low dosage of HMGB1 was administrated to evoke DC activation.

Tumor necrosis factor-α induced protein 8 like-2 (TNFAIP8L2, TIPE2) is the second member of tumor necrosis factor-α induced protein 8 (TNFAIP8) family, and it is recently defined as a novel protein expressed in lymphoid-derived and marrow-derived cells, thus manifesting a negative regulatory effect in the maintenance of immune homeostasis [12–15]. It was found that the onset of septic shock was dramatically accelerated and the process was exacerbated in TIPE2^−/−^ mice as compared with that in wild-type controls, suggesting that TIPE2 was directly responsible in preventing the occurrence of septic shock [12]. Furthermore, experiments *in vivo* demonstrated that TIPE2 might be involved in the immune regulation of T lymphocytes, and the decrease in TIPE2 expression on T lymphocytes could enhance peripheral T lymphocyte function after thermal injury [16]. Considering mature DCs are also essential in modulating the T lymphocyte proliferation and differentiation, we hypothesized that the TIPE2 might be related to the immune regulation mediated by DCs. Therefore, in the present study, we investigated the expression of TIPE2 in normal Balb/c murine DCs by Western blotting and reverse transcription polymerase chain reaction (RT-PCR), and also identified the potential effects of TIPE2 on DC maturation as well as its potential mechanism of regulating T-cell mediated immunity both *in vitro* and *in vivo*.

## RESULTS

### TIPE2 expression in DCs

**Figure 1:**
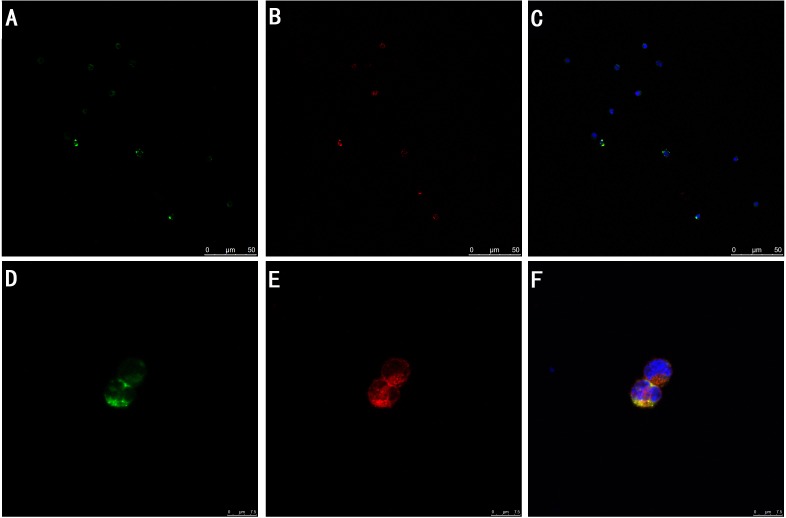
TIPE2 expression in DC cells (A-F) (*n* = 2) TIPE2 protein among FITC-positive DCs (green) **A.**, **D.** was determined by confocal laser scanning microscopy, and it was shown by antibodies indirectly labeled with DylightTM549 (red) **B.**, **E.** and stained cell nuclei (blue) **C.**, **F.** Representative photomicrographs showed that DylightTM549-positive cells (red) were shown among FITC positive DCs (green). Those double-stained cells were shown in yellow. E showed that TIPE2 was a cytoplasmic protein in DCs.

**Figure 2:**
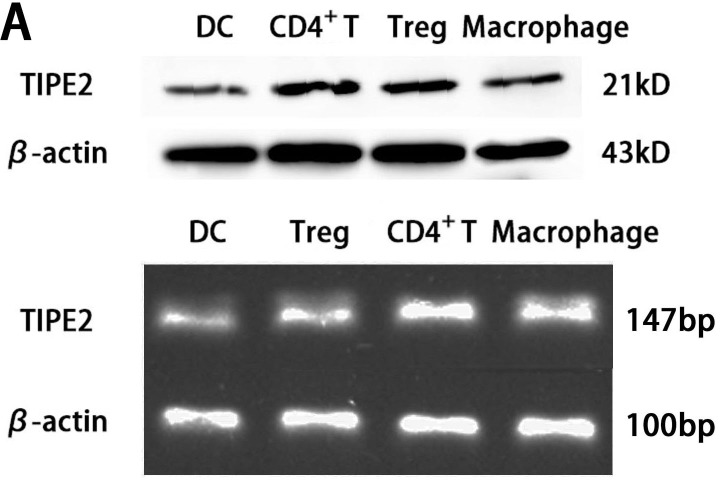

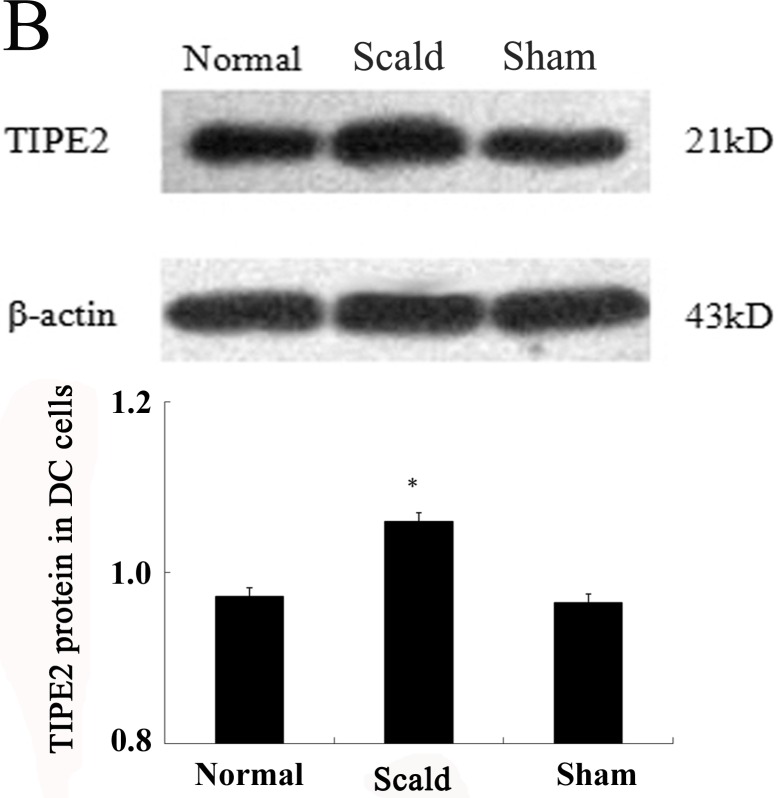
TIPE2 expressions in DCs from BALB/C mice (*n* = 5, in each group) **A.** Protein levels of TIPE2 in DCs were determined by Western blot. TIPE2 mRNA expression was measured by RT-PCR analysis in CD4^+^ T cells and DCs. Expression of β-actin served as an internal control. **B.** The protein expression of TIPE2 in DCs after scald injury. TIPE2 levels in DCs 24 h after scald injury were determined by Western blot analysis. The TIPE2 expression in DCs was significantly higher than that in sham group and normal group (*P* < 0.01). Statistical significance: *P < 0.01 scald group *versus* sham or normal group. All of the experiments were run for three times.

*In vitro* study, the expression of TIPE2 protein in DCs was investigated by means of confocal laser scanning microscopy. We stained fluorescein isothioctante (FITC)-labeled CD11c and DylightTM549-labeled TIPE2. As shown in Figure 1, green fluorescence could be observed on the cell surface of the DCs (Figure 1A, 1D) and red fluorescence in the cytoplasm of the DCs (Figure 1B, 1D), with their nuclei stained blue (Figure 1C, 1F). It was showed that TIPE2 was a cytoplasmic protein expressed in DCs. Gene expression of TIPE2 in DCs was assessed by RT-PCR, with β-actin as the internal standard. A band of the size of 147 bp was noticed as expected (Figure 2A). The expressions of TIPE2 mRNA in T cells, regulatory T cells (Treg), and macrophage, which are known to express a high level of TIPE2, were determined as a positive control. To further confirm the expression of TIPE2, it was measured at the protein level by Western blot analysis using the specific TIPE2 antibody, and clear bands with a molecular mass of approximately 21 kDa from DCs, CD4^+^ T cells, Treg, and macrophage were noted. The latter was used as the positive control (Figure 2A).

*In vivo* study, the protein levels of TIPE2 of DC were determined 24 h after scald injury. As shown in Figure 2B, TIPE2 levels were significantly elevated in the scald injured group compared with that of the normal controls (*P* < 0.01).

### TIPE2 prevented DC phenotypic maturation and cytokine expression

**Figure 3:**
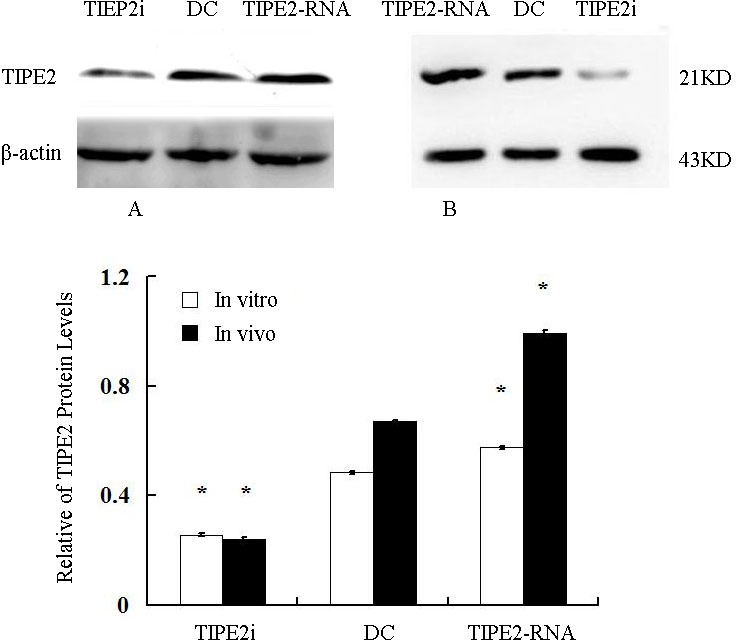
Relative abundance of TIPE2 protein levels *in vitro* (A) and *in vivo* (B) (*n* = 5, in each group) Representative Western blot gel showed relative abundance of TIPE2 protein levels in DCs (normal), siRNA-TIPE2 and TIPE2-RNA transfected DCs. Compared with the normal controls, *P < 0.05.

**Figure 4:**
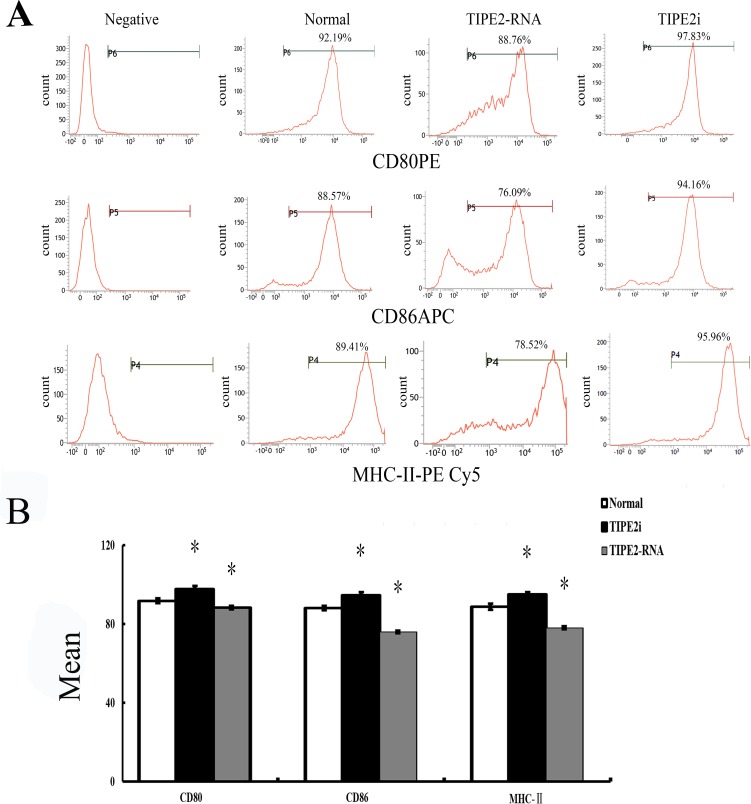

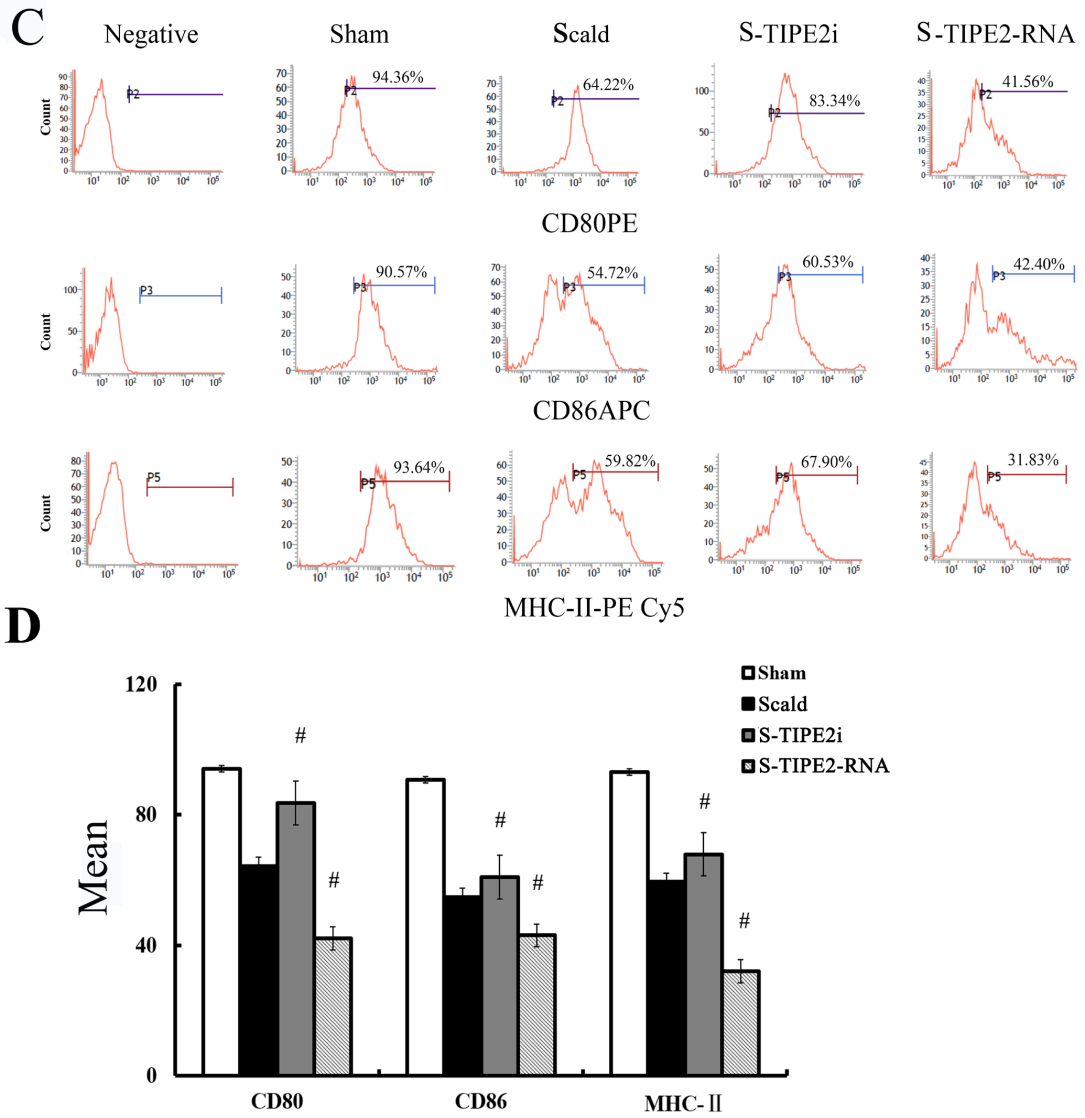
Changes in phenotype and percentage of DCs (*n* = 9, in each group) **A.** Representative flow cytometric analysis of CD80, CD86, and MHC-II expressions on DCs *in vitro*. **B.** Representative flow cytometric analysis of CD80, CD86, and MHC-II expression on DCs *in vivo*. **C.** *In vitro*, the percentage of DCs which expressed CD80, CD86, and MHC-II on DCs in siRNA-TIPE2 group was strongly increased compared with that in the normal group, while decreased in the TIPE2 over-expressed group (*P* < 0.01). **D.** In the scald injury group, the percentage of DCs which expressed CD80, CD86, and MHC-II was markedly decreased in TIPE2 over-expressed group, while elevated in the TIPE2 knockdown group (*P* < 0.01). Statistical significance: *in vitro*, *P < 0.01 as siRNA-TIPE2 or TIPE2-RNA group *versus* normal group; *in vivo*, ^#^*P* < 0.01 as siRNA-TIPE2 or TIPE2-RNA group *versus* scald group.

*In vitro,* our recent studies chose low dosage of HMGB1 to induce the activation of DCs, just like the role of LPS or TNF-α, which are considered to be important class of stimuli that evoke DCs to activation. In the current study, TIPE2 gene over-expressed or silenced by siRNA was used in this experiment (Figure 3). By Western blot analysis, TIPE2 protein level was markedly decreased in siRNA-TIPE2 (TIPE2i) transfected DCs, and significantly elevated in TIPE2-RNA transfected DCs compared with the normal controls (Figure 3, *P* < 0.05). To investigate the effect of TIPE2 in DC phenotypic maturation *in vitro*, DCs were stimulated with high mobility group box-1 protein (HMGB1) at 100 ng/ml for 24 h, and the resulting DCs were analyzed for phenotypic characteristics of activated DCs using untreated DCs as normal controls. As shown in Figure 4A, 4B, the percentage of DCs which expressed co-stimulatory molecules including CD80, CD86, and MHC-II was increased in comparison to the normal control group after TIPE2 gene silenced by siRNA (*P* < 0.05 or *P* < 0.01). Conversely, the percentage of DCs which expressed these molecules was markedly decreased when TIPE2 gene was over-expressed in DCs (P < 0.05 or P < 0.01). Furthermore, after scald injury, the percentage of DCs which expressed CD80, CD86, and MHC-II was significantly decreased in TIPE2 over-expressed mice, thus elevated in the TIPE2 knockdown mice (Figure 4C, 4D; *P* < 0.05 or *P* < 0.01).

**Figure 5:**
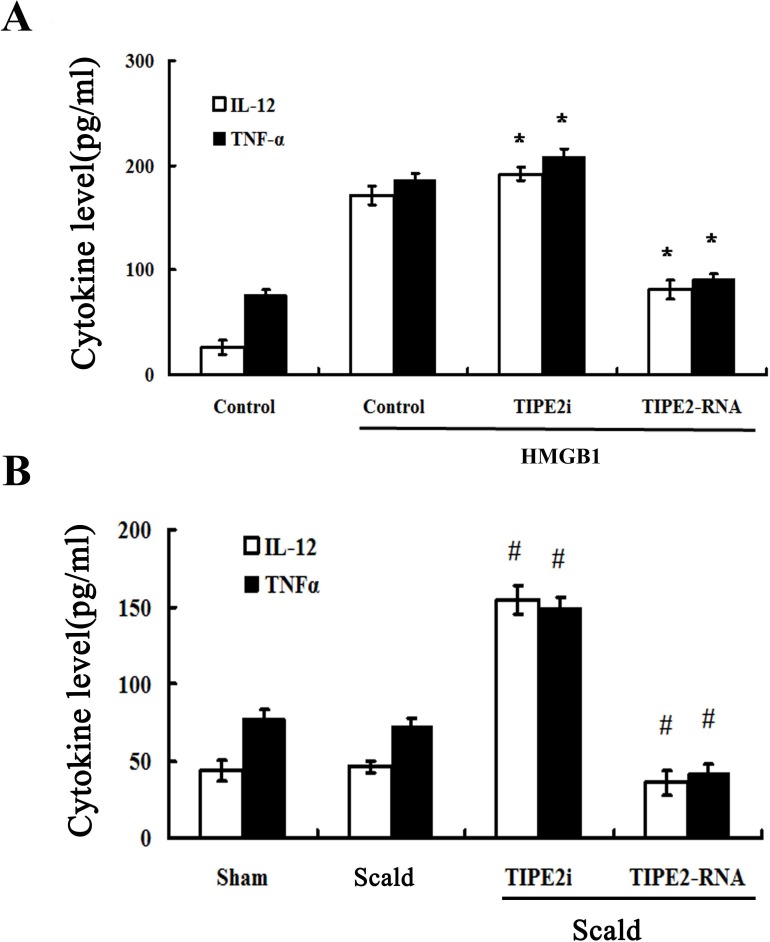
TIPE2 inhibited cytokine expression and secretion in DCs (*n* = 10, in each group) Immature DCs were cultured with the presence of 100 ng/ml HMGB1 or without HMGB1 (control) for 24 h. IL-12 and TNF-α levels were measured by ELISA *in vitro* **A.** or *in vivo* **B.**, respectively. Levels of IL-12 in the supernatants of DC were not markedly decreased after thermal injury, but TNF-α level was significantly lower in animals subjected to scald injury compared with sham injury after thermal injury. Treatment with siRNA to inhibit TIPE2 expression could significantly enhance the levels of IL-12 and TNF-α. TIPE2 over-expression could significantly decrease the levels of IL-12 and TNF-α. Results of experiments were shown as the mean ± SD. Statistical significance: *in vitro*, *P < 0.01 as siRNA-TIPE2 or TIPE2-RNA group *versus* control(HMGB1) group; *in vivo*, ^#^*P* < 0.01 as siRNA-TIPE2 or TIPE2-RNA group *versus* scald group.

To assess the release of cytokines reflecting DC maturation, interleukin (IL)-12, tumor necrosis factor (TNF)-α protein levels in supernatants in splenic DCs were measured by ELISA. *In vitro* study, when DCs were cultured in the presence of 100 ng/ml HMGB1 for 24 h, production of IL-12 and TNF-α by siRNA-TIPE2 transfected DCs were significantly enhanced, while a trend toward lower levels was noted in the TIPE2 up-regulated group as compared to the normal controls (Figure 5A; all *P* < 0.01). As shown in Figure 5B, levels of IL-12 and TNF-α in supernatants of DC were not elevated after 24 h of thermal injury, whereas TIPE2 knockdown group showed significant increase in levels of IL-12 and TNF-α (all *P* < 0.01), TIPE2 over-expressed group showed significant decrease in levels of IL-12 and TNF-α (all *P* < 0.01), and it was parallel to the expression of phenotypic molecules on the surface of DCs.

### TIPE2 prevented T cell proliferation and differentiation mediated by DCs

**Figure 6:**
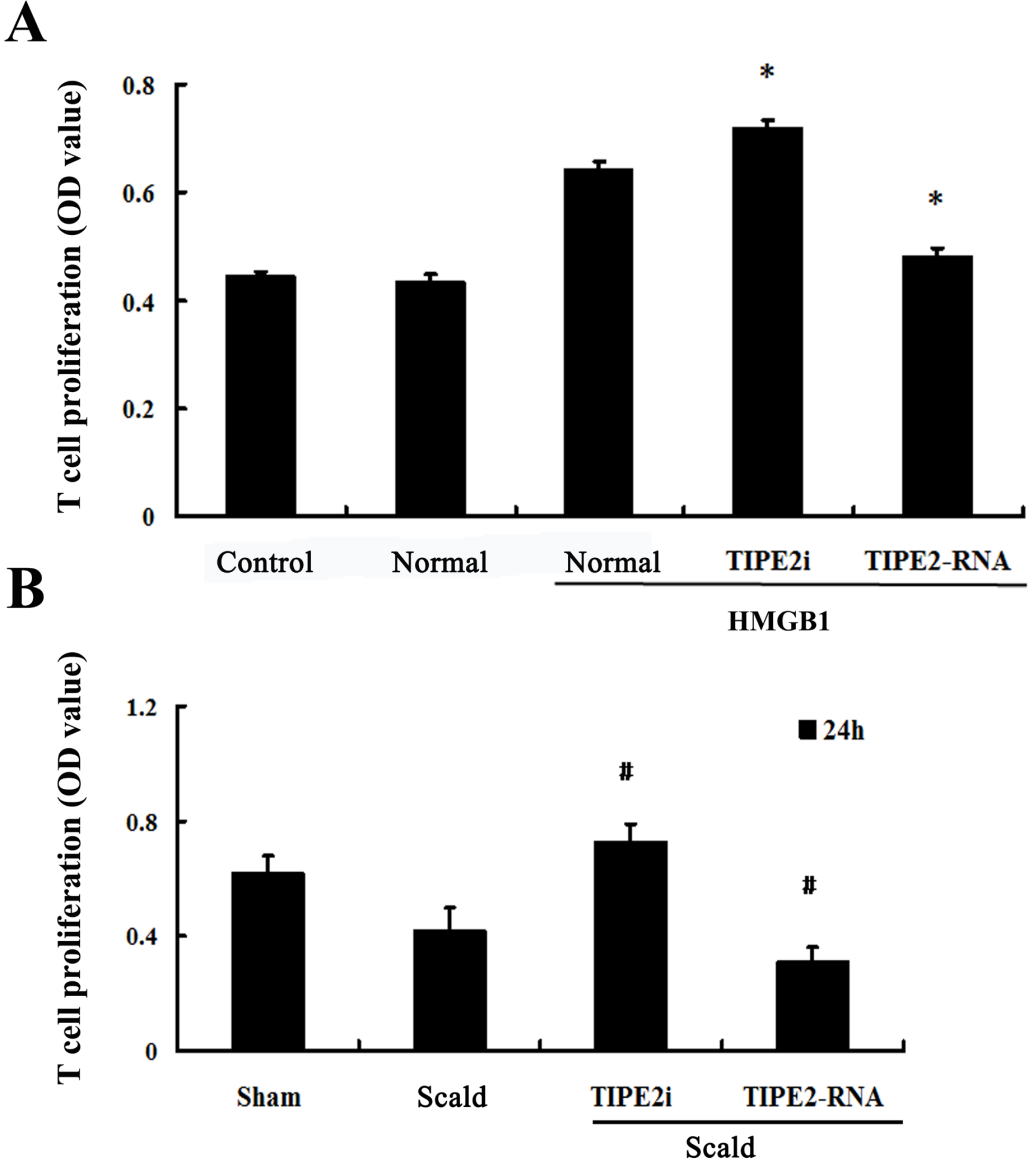
Effect of TIPE2 on the T-cell proliferation induced by DCs (*n* = 10, in each group) **A.** CD4^+^ T cells were incubated with ConA (5 μg/mL) for 18 h, then co-cultured with stimulated DCs (normal) at a ratio of 1:200 or cultured without DCs (control). **B.** After thermal injury, DCs were cocultured with CD4^+^ T cells at a ratio of 1:200. T cells were cocultured without DCs served as control. SiRNA-TIPE2 treatment could enhance T-cell proliferative activity response to Con A after scald injury. CCK-8 was used to measure T-cell proliferation activity. Results of experiments were shown as the mean ± SD. Statistical significance: *in vitro*, *P < 0.01 as siRNA-TIPE2 or TIPE2-RNA group *versus* normal (HMGB1) group; *in vivo*, ^#^*P* < 0.01 as siRNA-TIPE2 or TIPE2-RNA group *versus* scald group.

*In vitro*, HMGB1-treated DCs were analyzed for their capacity to stimulate the proliferation and differentiation of T cells in response to concanavalin A (Con A) in order to reflect functional maturation of DCs. T-cell proliferative activity and production of cytokines were determined. As shown in Figure 6A, the proliferation of T cells in response to Con A in the TIPE2-overexpressed group was significantly suppressed, but it could be enhanced by treatment with TIPE2 gene silence in DCs (*P* < 0.01). After 24 h of scald injury, T-cell proliferative activity was markedly down-regulated as compared with the sham injured group, lowest in the TIPE2 over-expressed group, whereas it was restored in the TIPE2 knockdown group (Figure 6B; *P* < 0.01).

**Figure 7:**
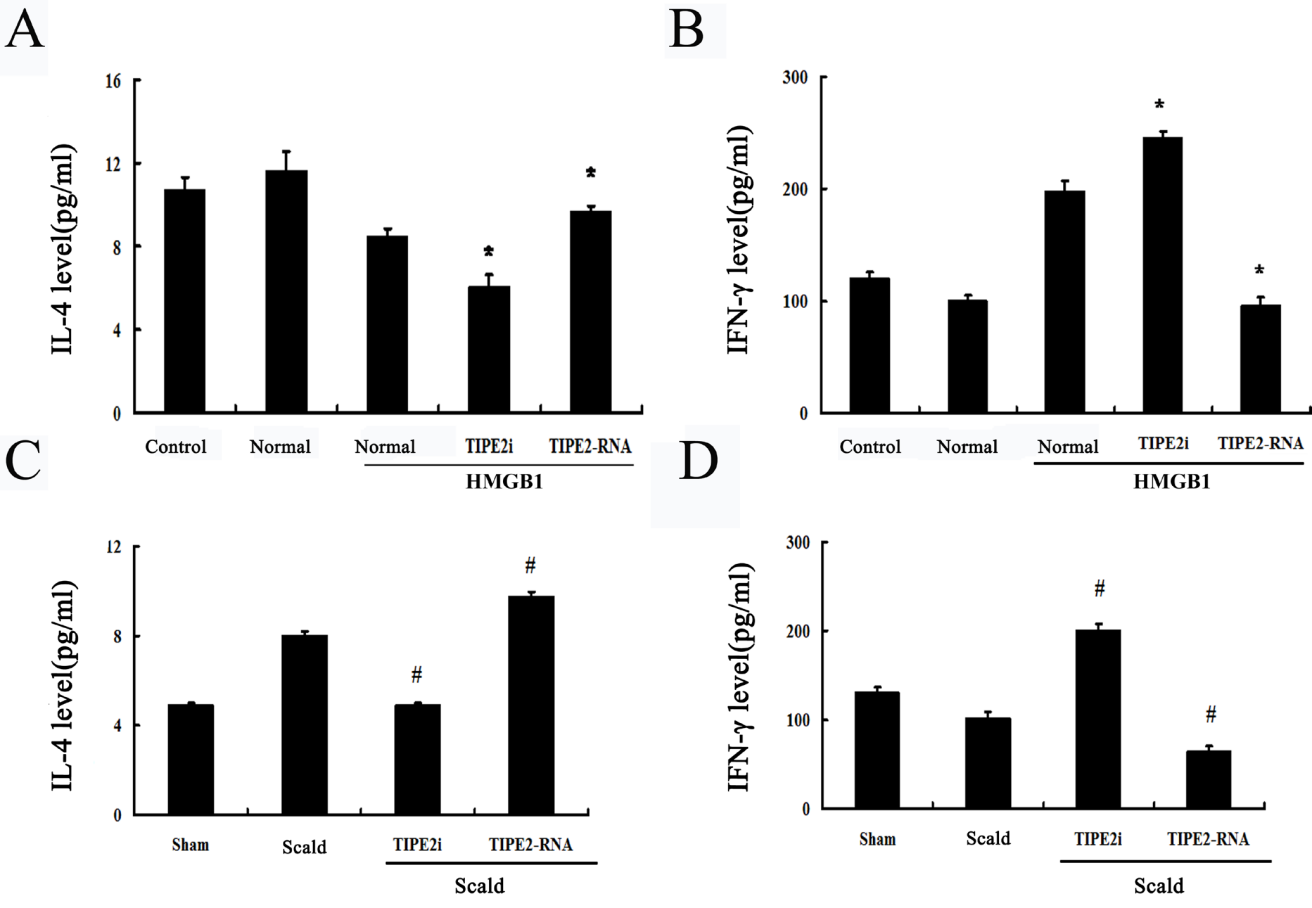
Effect of TIPE2 on the T-cell differentiation induced by DCs (*n* = 10, in each group) **A.** CD4^+^ T cells were incubated with ConA (5 μg/mL) for 18 h, then co-cultured with stimulated DCs (normal) at a ratio of 1:200 or cultured without DCs (control). IL-4 levels in culture medium were measured by ELISA. **B.** After thermal injury, DCs were cocultured with CD4^+^ T cells at a ratio of 1:200. IL-4 levels in culture medium were determined by ELISA to evaluate Th1/Th2 polarization. After thermal injury IL-4 levels produced by T cells response to Con A were markedly increased, whereas IFN-γ levels were markedly lowered. SiRNA-TIPE2 treatment could significantly revert elevated levels of IL-4 and lowered levels of IFN-γ after thermal injury. **C.** *In vitro*, IFN-γ levels in culture medium were determined by ELISA. **D.** *In vivo*, IL-4 levels in culture medium were determined by ELISA to evaluate Th1/Th2 polarization. Results of experiments were shown as the mean ± SD. Statistical significance: *in vitro*, *P < 0.01 as siRNA-TIPE2 or TIPE2-RNA group *versus* normal (HMGB1) group; *in vivo*, ^#^*P* < 0.01 as siRNA-TIPE2 or TIPE2-RNA group *versus* scald group.

**Figure 8:**
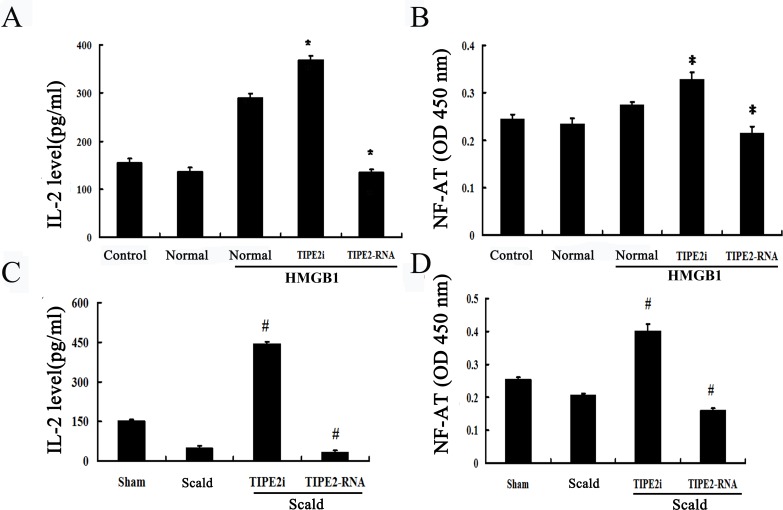
Effects of TIPE2 on IL-2 expression and intranuclear NF-AT activation in DCs in coculture experiments (*n* = 10, in each group) **A.** CD4^+^ T cells were incubated with ConA (5 μg/mL) for 18 h, then co-cultured with stimulated DCs (normal) at a ratio of 1:200 or cultured without DCs (control). IL-2 levels in the conditioned media were measured by ELISA. **B.** After thermal injury, DCs were cocultured with CD4^+^ T cells at a ratio of 1:200. The level of IL-2 in the conditioned media was measured by ELISA. **C.** *In vitro*, intranuclear NF-AT activity of T cells in the nuclear cell extract was measured by ELISA. **D.** *In vivo*, intranuclear NF-AT activity of T cells in the nuclear cell extract was measured by ELISA. Results of experiments were shown as the mean±SD. Statistical significance: *in vitro*, *P < 0.01 as siRNA-TIPE2 or TIPE2-RNA group *versus* normal (HMGB1) group; *in vivo*, ^#^*P* < 0.01 as siRNA-TIPE2 or TIPE2-RNA group *versus* scald group.

In the present study, culture supernatants were subsequently analyzed to observe changes in Th1 and Th2 cytokines. As shown in Figure 7A *in vitro*, levels of IL-4 produced by T cells with Con A stimulation was elevated in the TIPE2-overexpresed group, while interferon (IFN)-γ levels were lowered (all *P* < 0.05). However, DCs rendered TIPE2 gene silence significantly enhanced expression of IFN-γ and reduced expression of IL-4 of T cells in response to Con A (all *P* < 0.05). On the other hand, *in vivo*, we found a significant increase in IFN-γ/IL-4 ratio in T cells after TIPE2 gene silenced by siRNA in DCs, while a marked decrease in the ratio after over-expression of TIPE2 gene as compared with the scald injury group (Figure 7C, 8D; *P* < 0.01).

### TIPE2 down-regulated IL-2 expression and intranuclear nuclear factor of activated T cells (NF-AT) activation in T cells in coculture experiment

IL-2 is a potent T-cell growth factor that acts upon itself in an autocrine fashion. We examined the expression of IL-2 after CD4^+^ T cells were co-cultured with DCs. As shown in Figure 8A, IL-2 level was significantly lowered after TIPE2 over-expressed in DCs than that in the normal (HMGB1) group, whereas its expression was enhanced in the TIPE2 knockdown group (*P* < 0.01). In accordance with *in vitro* experiment, we found that IL-2 level was down-regulated after 24 h of thermal injury, while it was markedly up-regulated after TIPE2 gene silenced by siRNA in DCs (Figure 8C; *P* < 0.01).

To further characterize the signal pathway of T cell activation as promoted by DCs, we performed an experiment to assess NF-AT activation, which regulates transcription of the IL-2 cytokine. As shown in Figure 8B, NF-AT activation of splenic T cells was significantly up-regulated when TIPE2 gene of DCs prior silenced by siRNA (*P* < 0.05), while over-expression of TIPE2 in DCs could down-regulate its activation (*P* < 0.05). Furthermore, NF-AT activation was markedly enhanced in the TIPE2 knockdown group as compared with the scald injury group, while decreased in the TIPE2 gene over-expressed group (Figure 8D; *P* < 0.05).

## DISCUSSION

Dysfunction of specific immunity is a consequential phenomenon of burn injury, and it generally is believed that it is attributable to depression of T lymphocyte proliferation associated with the loss of function of the Th1 lymphocyte phenotype [17, 18]. It is our belief that further investigation into the mechanisms underlying the host immune suppression might contribute to formulate better therapeutic modalities for acute injury. TIPE2 is recently described as a novel gene independently required for maintaining immune homeostasis, constitutively expressed in various stages of macrophage, B and T lymphocytes, and it belongs to TNFAIP8 family [19–21]. It was also reported that TIPE2 in the porcine alveolar macrophages (PAMs) was significantly decreased at 12 h post-infection with porcine reproductive and respiratory syndrome virus (PRRSV). In addition to inflammatory diseases [21], TIPE2 is also an essential regulator in the pathogenesis of autoimmune diseases, such as systemic lupus erythematosus (SLE) and diabetic nephropathy (DN). In septic mice induced by lipopolysaccharide (LPS), septic shock was dramatically accelerated and exacerbated in TIPE2^−/−^ mice as compared to wild type controls. Using knockout, knockdown and gain-of-function approaches, it had been demonstrated that TIPE2 might be a novel negative regulator of immune function, and it was required in preventing hyper responsiveness and maintaining immune homeostasis [12].

In our previous study, we found that CD4^+^ T cells obtained from naive Balb/c mice positively expressed TIPE2, which was markedly increased at 24,48 and 72h after thermal injury, and the peak level appeared at 24 h after thermal injury [22]. In addition to CD4^+^ T cells, it has been proposed that DCs might play a central role in the initiation and control of the adaptive immune response in the peripheral lymphatic system, and studies in patients suffering from burns and subsequent sepsis have demonstrated a marked reduction in the number of splenic DCs and the percentage area of the spleen that was occupied by DCs [23, 24]. Uncontrolled inflammatory response and immunosuppressive response followed by tissue and organ dysfunction were also associated with DC loss and apoptosis after burn injury or septic challenge. Moreover, HMGB1, an important extracellular mediator of inflammation described recently, was proven to play an important role in DCs maturation and activation [11]. Our recent studies just chose low dosage of HMGB1 to induce the activation of DCs *in vitro*, similar to the effect of LPS or TNF-α, which was considered to be important mediators of stimuli that evoke DCs to activation.

In the present study, using confocal laser scanning, we firstly reported that TIPE2 protein was expressed in the cytosol of DCs, suggesting that TIPE2 might be of potential significance in DC mediated immune response. To further confirm the expression of TIPE2 in DCs, we determined the TIPE2 mRNA and protein levels in this study by RT-PCR and Western blotting, respectively. A band of the size of 147 bp and a clear band with a molecular mass of approximately 21 kDa from DCs were noted as expected. It is well known that complete activation of DCs acquires a mature phenotype, which is characterized by the up-regulation of MHC and T-cell co-stimulatory proteins on their cellular membrane essential for antigen presentation, showing a change in chemokine responsiveness, and production of a variety of inflammatory cytokines as well as chemokines [25–30]. With a thermal injury model, we observed the effect of TIPE2 on the DC maturation as well as activation at 24 h after thermal injury, while the potential role of TIPE2 in mediated immune function of DCs at various stages postburn injury should be studied in the further investigations. It was shown that the percentage of DCs which expressed the co-stimulatory molecules including CD80, CD86, and MHC-II expressions, as well as IL-12 and TNF-α levels were markedly increased in the TIPE2 knockdown group as compared with that of the scald injury group. On the contrary, the percentage of DCs which expressed those molecules were decreased after up-regulation of TIPE2 expression. *In vitro*, it was also demonstrated that silence of TIPE2 expression could result in marked increase of the percentage of DCs which expressed CD80, CD86, MHC-II, as well as IL-12 and TNF-α release in DCs. These findings suggested that TIPE2 appeared to be a potential negative regulator that could inhibit DC maturation both *in vitro* and *in vivo*.

Since TIPE2 knockdown enhances DC maturation, would it influence the capacity of matured DC in modulating cell-mediated immunity? To elucidate the potential role of TIPE2 on DC mediated immune response, we determined T-cell proliferation and differentiation induced by DCs. The results showed that DCs markedly enhanced T-lymphocyte proliferative response to T-cell mitogen in the TIPE2 knockdown mice after scald injury. It has been documented that DCs can direct specific immunity by inducing T cells to become effector, memory, or anergic T cells, or to undergo apoptosis [31]. Whether effector T cells take on a Th1 or Th2 phenotype is thought to be a function of the duration of the DC-T cell interaction, as well as the cytokine profile being produced by DC at the time of such interaction [32, 33]. In our *in vivo* study, TIPE2 expression knockdown in mice significantly increased the levels of Th1 cytokine (IFN-γ) and lowered the levels of Th2 cytokine (IL-4). Similarly, in the *in vitro* experiment, it was noticed that DC cells with TIPE2 knockdown significantly enhanced T-lymphocyte proliferation, and increased Th1 cytokine (IFN-γ) levels and decreased Th2 cytokine (IL-4) levels after being stimulated with HMGB1 (100 ng/ml). Conversely, we found that T-lymphocyte proliferation induced by DCs and IFN-γ release were markedly reduced, but IL-4 level was elevated in over-expressing TIPE2 DC cells. Taken together, these results indicated that TIPE2 might suppress the capacity of matured DC in modulating T cell-mediated immunity, through a remarkably decreased capacity for presenting antigen to T cells for the stimulation of cell proliferation as well as differentiation. In addition, our data confirmed that treatment with TIPE2-siRNA to inhibit TIPE2 expression after thermal injury, the function of mature DC and T-cell activity were restored, accompanied by shifting of T cells to Th1.

We next examined IL-2 levels in culture supernatants obtained from DC-T cell coculture, and a significant increase was found in the TIPE2 knockdown group, with a marked decrease in the over-expressing TIPE2 group as compared with the scald injury group. IL-2 is a potent T-cell growth factor, and a decreased IL-2 production may lead to the depression of T-cell function after acute insults. In the current experiment, similar to the *in vivo* results, it was noted that IL-2 production was markedly reduced when naïve T cells were co-cultured with DC over-expressing TIPE2. Engagement of T cell receptors results in the activation of various signaling pathways, the most important of which is calcium-induced activation of the phosphatase and calcineurin, which dephosphorylates NF-AT [18, 30]. The NF-AT transcription factor family represents an important group of regulators, playing a central role in regulating transcription of IL-2 and coordinating cytokine expression [34–37]. Our preliminary experiments showed that TIPE2 silenced by siRNA in T cells *in vivo* specially influenced the NFAT activity, thus we focused on NFAT in the recent study, through which we hope to enhance understanding of the molecular mechanism of TIPE2 in immunological regulation. In the current study *in vivo*, recombinant lentiviruses that carry the TIPE2 siRNA or TIPE2 RNA were injected intraperitoneally to mice before scald injury, DCs were obtained from these mice in which TIPE2 was silenced or up-regulated, thus CD4^+^ T cells were isolated from normal BALB/c mice. Our results showed that when naïve T cells were co-cultured with DCs for 72 h at a ratio of 1:200 resulted in an increase in NF-AT activity of CD4^+^ T cells in response to ConA in the TIPE2 knockdown group, and over-expression of TIPE2 in DC cells reduced NF-AT activity of CD4^+^ T cells after thermal injury. Therefore, TIPE2 might inhibit NF-AT signaling, and it is the potential signal pathway in regulating cytokine expression of T lymphocytes induced by matured DCs. Nevertheless, this study has limitations. T cells in these studies are activated in an antigen independent system, in which the mitogen, Con A provides the first activation signal, and the DCs provide only co-stimulatory molecules as the second signal. Therefore, in a further study, an allogenic MLR or an antigen specific model should be used to better study the effects of TIPE2 on the antigen presenting capacity of DCs and on the generation of adaptive immune responses, relevant in thermal injury.

In summary, our data indicate that TIPE2 can suppress the burn or HMGB1-induced the percentage of DCs which expressed CD80 as well as MHC class II and IL-12 production** both *in vivo* and *in vitro*, thereby resulting in the decrease of IL-2 dependent T-cell proliferation and shifting of Th1 to Th2. TIPE2 appears to be an essential negative regulator involved in DC maturation as well as DC-mediated immune response.

## MATERIALS AND METHODS

### Ethics statement

All experimental manipulations were performed in accordance with the National Institute of Health Guide for the Care and Use of Laboratory Animals, with the approval of the Scientific Investigation Board of the Chinese PLA General Hospital (No. SYXK2012-0014), Beijing, China.

### Medium and reagents

Water soluble tetrazoliun-8 (WST-8) assay kits were purchased from Huayasi Biotechnology Co., Beijing, China. Con A was purchased from Sigma, St. Louis, MO. Recombinant HMGB1 was purchased from R&D System, Inc, Minneapolis, USA. Antibodies used for flow cytometry analysis, including (PE-Cyanine5)-conjugated anti-mouse MHC-II were purchased from eBioscience, San Diego, CA. PE-conjugated anti-mouse CD86, allophycocyanin (APC)-conjugated anti-mouse CD80, FITC-conjugated goat anti-mouse CD11c was purchased from Miltenyi Biotec GmbH, Bergisch Gladbach, Germany. Rabbit anti-mouse TIPE2 polyclonal antibodies were purchased from America Basic GENE Associate Bioscience Inc., Chicago, IL. Total RNA isolation system and reverse transcription system were purchased from Invitrogen, California, CA, and Promega, Madison, WI. SYBR Green PCR Master MIX was purchased from Applied Biosystems, Foster City, CA. Dendritic Cell Isolation Kit was purchased from Miltenyi Biotec GmbH, Bergisch Gladbach, Germany. ELISA kits of IL-12p40, IL-2, IL-4, IFN-γ, and TNF-α were purchased from Biosource, Worcester, MA. Nuclear extract and NF-AT assay kits were purchased from Active Motif, Carlsbad, CA.

### Animal thermal injury model

All male BALB/c mice had free access to water but were fasted overnight, and the hair on the animals’ backs was removed with 20% sodium sulfide before the experiment. Mice were anesthetized with aether, and subsequently the exposed back skin of the mice was immersed into 94°C water for 7 s. Sham-injured mice were subjected to all of the procedures except the temperature of the bath was at room temperature [38]. Lactated Ringer solution was administered intraperitoneally for initial resuscitation 6 h after injury, and then 1.0 ml was administered at 12 h, respectively, after scald injury.

### Experimental design

One hundred and fifty-six mice were randomly divided into 4 groups as follows: sham group (39 mice), scald group (39 mice), scald with siRNA-TIPE2 treatment group (39 mice), and scald with TIPE2-RNA treatment group (39 mice). In addition, 39 mice were taken to serve as normal controls (the main parameters determined in the current study were found to be highly constant in 39 mice of sham burn animals, so the results were not shown). Recombinant lentiviruses that carry the TIPE2 siRNA or TIPE2 RNA were injected intraperitoneally to mice 10 days before scald injury, respectively in scald + siRNA-TIPE2 group and in scald + TIPE2-RNA group. Animals of all groups were sacrificed at 24h after scald injury, all spleen samples were used to procure DCs and CD4^+^ T cells. *In vitro*, DCs were obtained and transfected with recombinant lentiviruses that carry the TIPE2 siRNA or TIPE2 RNA for 48h, subsequently stimulated with HMGB1 for 24h and the normal DCs were used as the controls. The viability of DCs was about 89% as determined directly at 24 hours after the transfection process by means of trypan blue exclusion.

### Isolation of splenic DCs and T lymphocytes

Spleens were obtained from normal BALB/C mice and burn mice, and they were teased in 5 ml RPMI 1640. Mononuclear cells were isolated with the use of Ficoll-Paque density gradient centrifugation, and then DCs were isolated from them. DCs were stained using CD11c MicroBeads (10 μl/10^7^ total cells), a MiniMACS™ Separator with a positive selection MS column by following the manufacturer's instructions. The pellets of selected DCs were obtained by centrifugation (1500 rpm, 10 min), then the supernatant was discarded, and the cells were then cultured in RPMI medium1640, supplemented with 10% heat-inactivated FCS. Purity of DCs was greater than 93% as determined by flow cytometry.

CD4^+^ T cells were isolated with CD4^+^ T cell Isolation Kit. Mononuclear cells were stained with biotin-antibody cocktail (10 μl/10^7^ total cells) and incubated for 10 min at 4°C. They were then magnetically labelled with anti-biotin microbeads (20 μl/10^7^ total cells), incubated for 15 min at 4°C, and harvested through a negative selection MS column (purity of purified CD4^+^ T cells >90%).

### Flow cytometric analysis

*In vitro*, DCs were obtained and transfected with recombinant lentiviruses that carry the TIPE2 siRNA or TIPE2 RNA for 48h, subsequently stimulated with HMGB1 for 24h and the normal DCs were used as the controls. DCs (5 × 10^5^) in 100 μl of PBS 5%FCS and 0.1% sodium azide (staining buffer) were reacted for 15 min at 4°C with PE-conjugated IgG specific for CD86, or with APC-conjugated anti-mounse CD80, PE-Cyanine5- conjugated anti-mounse MHC-II, FITC-conjugated anti-mounse CD11c. Monoclonal antibodies were added to the appropriate tubes and maintained at 4°C for 30 min. Finally, cells were washed and fixed in 1% formaldehyde/PBS and analyzed with FACSCalibur using CellQust software.

### Western blot and confocal microscopy analysis

Western blot was performed to determine the expression of TIPE2 in DCs. Total protein concentration of samples was measured using bicinchoninic acid protein assay kit. 50 μg of total protein/sample was loaded on 8% Tris-HCl sodium dodecyl sulfate-polyacrylamide gel, and the products were electrically transferred to an immobilon polyvinylidene difluoride membrane. After blocking with 10% skim milk overnight at 4°C, the membrane was incubated for 4 h at room temperature with anti-TIPE2 polyclonal antibody, followed by peroxidase-labelled affinity secondary antibody for 1 h at room temperature. After three washings, the membrane was determined by using the ECL plus chemiluminescence kit.

After treatment with HMGB1 for 24 h, DCs were washed with PBS for three times, and fixed with 4% paraformaldehyde in PBS for 20 min, then permeabilized with 0.02% Triton X-100 for 20 min at room temperature. Sections were preblocked with 1% bovine serum albumin in PBS for 30 min and stained with anti-TIPE2 antibody (1:200) overnight at 4°C. After being washed in PBS for three times, DCs were stained with goat anti-IgG (Dylight TM549) as the second antibody for 1 h at room temperature followed by 3×PBS washes. DCs after being stained with intracellular proteins were stained with FITC-labelled anti-CD11c monoclonal antibody. After being washed, the nuclei were stained with 4′,6-diamidino-2- phenylindole (DAPI) for 5 min. The cells were observed with a laser scanning confocal microscope.

### Extraction of total RNA and RT-PCR

Total RNA was extracted from DCs (2×10^6^) using TRIZOL kits as we described previously [39, 40]. The concentration of purified total RNA was determined spectrophotometrically at 260 nm. After removal of potentially contaminating DNA with DNase I, 1 μg of total RNA from each sample was used for RT with an oligo dT and a Superscript II to generate first-strand cDNA. The level of β-actin mRNA was also designed as an internal control for each sample. Primers for TIPE2 were forward 5′;-TCAGAAACATCCAAGGCCAGAC-3′; and reverse 5′;-CGGACCGACCAGCCATTTTAC-3′; [41]. For β-actin, we used the following primers: forward5′;-ATTGGCAATGAGCGGTTCCG-3′;, reverse 5′;- AGGGCAGTGATCTCCTTCTG-3′;. The thermal cycling conditions were as follows: 95°C for 15 min, followed by 35 cycles of denaturation, annealing and amplification (94°C for 40 s, 50°C for 40 s, 72°C for 30 s), and a final extension period of 5 min at 72°C. All samples were run in quadruplicates.

### RNA interference

Small interference RNA to TIPE2 (siRNA) or over-expression RNA to TIPE2 (TIPE2 RNA) was synthesized by Genchem Co., Shanghai, China. The DNA target sequence for siRNA-TIPE2 was 5′;-GAAGTGAAACTCAGGTCCG-3′; [12], and the scrambled control RNA was 5′;-TTCTCCGAACGTGTCACGT-3′;. To knock-down or up-regulate TIPE2 expression, DCs were infected with recombinant lentiviruses that carry the TIPE2 siRNA or TIPE2 RNA. Transfection of recombinant lentiviruses was performed according to the manufacture's instruction in Lentiviral Vector Particle. The MOI of lentivirus carried the TIPE2 siRNA or TIPE2 RNA was used to transduce DC was 2E+9 TU/ml, 5E+8 TU/ml, respectively, and the total dose of lentivirus was used to transduce DC *in vitro* or administered to mice *in vivo* was 1E+8 TU/ml. *In vivo*, male mice was individually injected with 3 ×10^7^ TU lentivirous vector *via* the tail vein. The transduction efficiency for DC *in vitro* and *in vivo* was both greater than 60%. *In vitro*, 3 days later, the efficiency of knock-down or over-expression was checked by Western blot for TIPE2; *in vivo*, TIPE2 expression was determined on day 10 after recombinant lentiviruses were injected intraperitoneally to mice.

### CCK-8 assay

*In vitro*, DCs were stimulated for 24 h with 100 ng/ml HMGB1, then they were thoroughly washed before co-culture with Purified CD4^+^ T cells. T cells were incubated with Con A (5 μg/ml) for 18 h, then co-cultured with stimulated DCs in the cell-ratio of 1:200. *In vivo*, CD4^+^ T cells were isolated from naïve mice and treated with 5 μg/ ml Con A. Mixed cells were plated into 96-well flat bottom plates at 1×10^5^ cells/well and cultured in a medium containing 10%FCS at 37°C in 5% CO_2_ in humidified air for 72 h. Thereafter, 10 μl CCK-8 solution was added to each well, the optical density of T-cell proliferation activity was measured by the use of a microplate reader.

### Cytokine and NF-AT activity measurements with ELISA

The nuclear cell extract was prepared. The levels of IL-2, IL-4, IFN-γ, TNF-α, IL-12p40 and the active form of NF-AT contained in nuclei was measured with ELISA.

### Statistical analysis

Data was represented as mean ± standard deviation (SD). Data sets were examined by one-way ANOVA, and individual group means were then compared with Student paired t test. All statistical tests were two sided and a P value of 0.05 or less was considered to indicate statistical significance.
